# Conspecific Presence Promotes Social Buffering, Restores Social Reward, and Enhances Spatial Navigation in a Ketamine‐Induced Model of Schizophrenia in Mice

**DOI:** 10.1111/ejn.70359

**Published:** 2025-12-17

**Authors:** Rayan Fidel Martins Monteiro, Marcos Vinícius Lebrego Nascimento, Klinsmann Thiago Lima, Valdina Solimar Lopes Cardoso, José Ramon Gama Almeida, Wellington Junior Taisho Nagahama Costa, Bruno Eduardo Godinho Teixeira, Vinicius Teles Shirakura, Soraya Amin Souza, Juliana Silva Cassoli, Gilmara de Nazareth Tavares Bastos

**Affiliations:** ^1^ Laboratory of Neuroinflammation, Institute of Biological Sciences Federal University of Pará Belém Pará Brazil; ^2^ Laboratory of Reproductive and Development Biology Federal University of São Paulo São Paulo Brazil

**Keywords:** anxiety‐like behavior, ketamine, schizophrenia, social buffering, social cognition

## Abstract

Despite extensive research on the effects of enriched environments in mouse models of psychiatric disorders, the role of social context remains poorly explored. Therefore, we assessed the impact of a conspecific during the evaluation of negative and cognitive symptoms in a mouse model of schizophrenia (SCZ). Male C57BL/6J mice received daily injections of ketamine (25 mg/kg) or vehicle. Behavioral testing was conducted with mice either alone or in dyads of varying familiarity. Anxiety‐like behavior and habituation were assessed using the 3D maze test (3DM). Social reward was measured with a social conditioned place preference test. Finally, episodic memory was evaluated using an object recognition memory test (ORMT). Ketamine induced anxiogenic‐like behavior and impaired habituation, social reward, and spatial memory. In contrast, the presence of a conspecific induced anxiolytic‐like behavior, accelerated habituation, and restored social reward. Additionally, greater dyad familiarity led to better performance in the 3DM. Conversely, the presence of a conspecific did not rescue cognitive deficits in the ORMT; however, spatial navigation in the 3DM was improved. These results support that the presence of a conspecific induces social buffering and promotes prosocial behavior. Therefore, this highlights a therapeutic effect modulated by social context and introduces a new model to evaluate social cognition in a mouse model of SCZ.

## Graphical abstract

Conspecific presence during the tasks induces exposure‐type social buffering (i.e., anxiolytic‐like behavior and faster habituation), recovers social reward and improves spatial navigation in a mouse models of schizophrenia induced by ketamine.

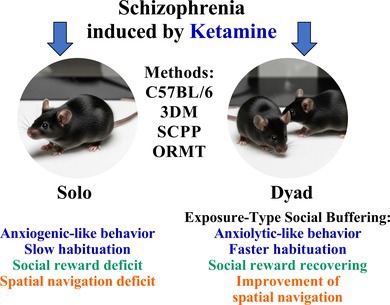

**Abbreviations**
3DM: 3D maze test
C: groups that performed dyad entry, where one dyad was from a different cage and the same pharmacological treatment
D: groups that performed dyad entry, where the dyad was from the same cage and the same pharmacological treatment
DC: groups that performed dyad entry, where one dyad was from a different cage and the same pharmacological treatment, and the dyads were always different over the 5 days
ETSB: exposure‐type social buffering
KET: ketamine group(s)
KET‐C: ketamine group(s) that performed dyad entry, where the dyad was from a different cage and the same pharmacological treatment
KET‐D: ketamine group(s) that performed dyad entry, where the dyad was from the same cage and the same pharmacological treatment
KET‐P: ketamine group(s) that performed dyad entry, where the dyad was from the same cage and different pharmacological treatments
KET‐S: ketamine group(s) that performed solo entry
LRM: location recognition memory
ORM: object recognition memory
ORMT: object recognition memory test
P: groups that performed dyad entry, where the dyad was from the same cage and different pharmacological treatments
S: groups that performed solo entry
SC: groups that performed dyad entry, where one dyad was from a different cage and the same pharmacological treatment, and the dyads were the same over the 5 days
SCPP: social conditioned place preference
VHC: vehicle group(s)
VHC‐C: vehicle group(s) that performed dyad entry, where the dyad was from a different cage and the same pharmacological treatment
VHC‐D: vehicle group(s) that performed dyad entry, where the dyad was from the same cage and the same pharmacological treatment
VHC‐P: vehicle group(s) that performed dyad entry, where the dyad was from the same cage and different pharmacological treatment
VHC‐S: vehicle group(s) that performed solo entry

## 1 Introduction

Characterized as a mental disorder, schizophrenia (SCZ) is systematically divided into three groups of symptoms: positive, negative, and cognitive. Positive symptoms, especially hallucinations and delusions, are essential for diagnosing this disease and can be mitigated with anti‐psychotic drugs. On the other hand, negative and cognitive symptoms are targets of psychological interventions. Negative symptoms are characterized by alterations in emotional, motivational, and sociability domains, whereas cognitive symptoms are characterized by deficits in learning, memory, attention, and executive functioning (Kesby et al. 2018).

Kovacs et al. (2018) reported that the healthcare costs of SCZ in Europe are associated with hospitalization, symptoms (especially negative and cognitive), age, persistence, and adherence. Therefore, those issues could be directly mitigated by an increase in non‐pharmacological approaches (Kovacs et al. 2018).

In pre‐clinical trials with non‐pharmacological interventions, such as enriched environment, promising outcomes have been achieved in pharmacological (Huang et al. 2021; Koseki et al. 2012; Xu et al. 2022), neurodevelopment (Bator et al. 2018; Zhu and Grace 2021), and genetic (Burrows et al. 2015; Harb et al. 2021; McOmish et al. 2008) models of SCZ. Similarly, in humans, an enriched environment approach in early life promotes better long‐term outcomes associated with the prevention of SCZ (Raine et al. 2003). Therefore, environmental modulation emerges as an essential feature or tool to promote quality of life in patients with SCZ.

Social deficits are a common factor in a wide range of psychiatric disorders. Thus, the social aspect of the environment emerges as a fundamental target for treating negative and cognitive symptoms in SCZ. Therefore, we investigated the presence of a conspecific animal during the behavioral manifestation of negative and cognitive symptoms in mice. The use of ketamine to induce SCZ‐like behavior has been identified as a good model for inducing social deficits (Hazani et al. 2022). The presence of a conspecific animal induces social buffering, which is the relief from an emotional stress status caused by a socially enriched environment. The social buffering can be defined in two different types: Exposure‐type social buffering (ETSB), which is “phenomena in which stress of the subject is ameliorated when the subject is exposed to distressing stimuli along with a conspecific animal(s)”; and housing‐type social buffering, which is “phenomena in which recovery from adverse alterations induced by previously distressing stimuli is led by subsequent co‐housing with a conspecific animal(s)” (Kiyokawa et al. 2007). Additionally, other studies suggest that the presence of a conspecific can improve episodic memory in naïve rats (de Franca Malheiros et al. 2020), in C57BL/6 mice, and in a mouse model of autism spectrum disorder (BTBR mice) (Lipina and Roder 2013). However, the evaluation of social cognition during the presence of another conspecific is an issue that is unexplored in murine models of psychiatric disorders.

Therefore, in this work, we investigated if the presence of a conspecific animal could: induce ETSB (evaluating the anxiety‐like behavior and habituation); modulate the social cognition (evaluating the social reward); or modulate the episodic memory (evaluating the object novelty recognition memory) in a mouse model of SCZ induced by ketamine.

## 2 Material and Methods

### 2.1 Animals and General Treatments

The experiments were conducted using 108 male C57BL/6 mice (8–10 weeks old; 20–30 g) housed randomly with 3–4 animals/cage (floor: 451 cm^2^), with sawdust and one cardboard tunnel per cage, at 22 ± 2°C under a 12/12 h light/dark cycle, with access to food and water ad libitum. Mice were obtained from Evandro Chagas Institute (Belém, Pará, Brazil). All animal procedures described in this work were reviewed and approved by the animal ethics committee from the Federal University of Pará (CEUA‐UFPA No. 69092809923). All behavioral tests and treatments were performed at least 1 week after the animals arrived in the laboratory. During this period, the animals were marked with an auricular punch under local anesthetic (Lidocaine), with a maximum of one punch per animal for individual discrimination. All animal procedures were always performed during the light phase of the cycle. At the end of the assays, all animals were euthanized individually with a lethal dose of xylazine and ketamine solution in a different room.

### 2.2 Drugs and Treatments

All animals were treated (i.p.) with saline solution (NaCl 0.9%) (vehicle) or 25 mg/kg of ketamine (*Syntec*) diluted in saline solution (5 mg/mL). All animals were treated with a 0.3 mL syringe in a volume of 5 mL/kg. All animals received a maximum of one injection per day in a separate room from the acclimation room.

### 2.3 3D Maze (3DM)

#### 2.3.1 Apparatus and Parameters

The 3DM (8‐arms radial maze variation) apparatus consists of an adaptation in “raised arms” configuration for assessing anxiety‐like behavior (Ennaceur et al. 2006). The maze used here consists of an MDF apparatus, featuring a central octagonal platform measuring 11.2 cm on each side at a height of 60 cm above the floor, 8 rectangular “bridges” measuring 15.2 cm × 11.2 cm, and 8 rectangular “arms” measuring 35 cm × 11.2 cm. The bridges form an angle of 140° in relation to the surface of the central platform. At the beginning of each bridge, there is a small square wall (5 cm × 5 cm) to prevent passage from one bridge to the other. In this configuration, the arms are higher than the central platform. Furthermore, at the end of each arm, there is a spatial identification plate (20.2 cm × 11.2 cm) (Figure S1A).

The main parameters measured in this test were: time on the arms; time on the center; number of arm entries; number of non‐visited arms; and number of approaches, which was considered when the animal in the center entered the bridges with its 2 front paws and then returned to the center.

#### 2.3.2 3DM Test Performing for Anxiety‐Like Behavior and Habituation Assessing

The animals were treated with vehicle or ketamine for 7 days. From the 4th to the 7th day, 20 min after the treatment, the animals were tested in 3DM once a day for 12 min (Figure S1B). All animals started the test on the central platform and were recorded using a digital camera. Each entry was considered when the animals crossed with their 4 paws on a zone (Central, Bridges, or Arms). Between each test, the maze was cleaned with 70% alcohol and wiped with tissue paper. The test was performed under 250 lx. The animals were separated into eight groups according to pharmacological treatment and the entry form on 3DM. Thus, there were two pharmacological treatments: vehicle (VHC) and ketamine (KET); and four entry forms: solo (S), dyad from the same cage and same pharmacological treatment (D), dyad from different cages and the same pharmacological treatment (C), and dyad from the same cage and different pharmacological treatment (P) (Figure S1A). In the C groups, the dyads were the same over the 4 days. All groups had six animals.

#### 2.3.3 3DM Test Performing to Discriminate C Groups

The animals were treated with ketamine for 8 days. From the 4th to the 8th day, 20 min after the treatment, the animals were tested in 3DM once a day for 10 min (Figure S1C). All animals began the test on the central platform and were recorded using a digital camera positioned above the apparatus. Each entry was considered when the animal crossed with its 4 paws on a zone (Central, Bridges, or Arms). Between each test, the maze was cleaned with 70% alcohol and tissue paper. The test was performed under 250 lx. All animals were treated with ketamine and entered into dyads from different cages and the same pharmacological treatment (C). However, the groups were separated according to dyad familiarity throughout the test: in one group, the dyads remained the same for the 5 days (SC), and in the other, the dyads were always different for the 5 days (DC). The SC and DC groups had six animals.

### 2.4 Social Conditioned Place Preference (SCPP)

The SCPP apparatus consisted of three transparent acrylic boxes, arranged side by side, connected by circular holes of 4 cm in diameter at 1 cm from the floor and in the center of the lateral walls. The two lateral boxes had a 25 × 25 cm area and a 17 cm height, and the one central box had a 25 × 10 cm area and a 13 cm height. All three boxes were kept in a polystyrene foam box to avoid visual and spatial cues (Figure S1D). The animals were treated with vehicle or ketamine for 4 days. On the 2nd day, 20 min after the treatments, the animals were habituated in the apparatus for 20 min. On the 3rd day, all animals were socially isolated for 24 h in a different cage with wood pellets, a cubic polystyrene (2 cm^3^), and food and water available ad libitum. Finally, on the 4th day, immediately after social isolation and 20 min after the treatments the animals were tested in the SCPP apparatus for 20 min, where one lateral box was covered with sawdust and one piece of a cardboard tunnel (social place: standard bed, to which the animals had been habituated to since acclimation), and the other lateral box was covered with wood pellets and one cubic polystyrene (2 cm^3^) (non‐social place: similar to the conditions in which the animal was conditioned on the 3rd day). On the 2nd and 4th days, all animals began the test in the central box and were recorded using a digital camera positioned above the apparatus (Figure S1E). The animals were separated into 4 groups according to pharmacological treatment and the entry form on the SCPP apparatus. Thus, there were two pharmacological treatments: vehicle (VHC) and ketamine (KET); and two entry forms: solo (S) and dyad from the same cage and same pharmacological treatment (D). All groups had 6 animals. The test was performed under 100 lx. The main parameters measured in this test were: time spent in social and non‐social boxes; and number of transitions, which was the frequency of crosses between boxes. This assay was based on other works in the literature (Dolen et al. 2013; Panksepp and Lahvis 2007; Ross et al. 2024).

### 2.5 Object Recognition Memory Test (ORMT)

The ORMT apparatus consisted of two transparent acrylic boxes, side by side, connected by circular holes of 4 cm in diameter, located 1 cm from the floor and in the center of the lateral walls. One box had a 25 × 25 cm area and 17 cm height (large box), and the other box had a 25 × 10 cm area and 13 cm height (small box). All two boxes were kept inside a polystyrene foam box. Outside and next to the two boxes, there was a wooden all on one of the sides of the apparatus to serve as a spatial cue (Figure S1F). The animals were treated with vehicle or ketamine for 6 days. On the 3rd day, 20 min after the treatments, the animals were introduced to the small box for 10 min (habituation). On the 4th day, 20 min after the treatments, the animals were introduced to the small box for 10 min, where there were two identical objects in the large box in the distant corners from the small box. On the 5th day, 20 min after the treatments, the animals were introduced to the small box for 10 min, where one object was displaced to one of the closer corners of the small box, and the other object stayed in the same corner to assess location recognition memory (LRM). Finally, on the 6th day, 20 min after the treatments, the animals were introduced to the small box for 10 min, where the displaced object was changed to a different object for assessing object recognition memory (ORM) (Figure S1G). The animals were separated into four groups according to pharmacological treatment and the entry form on the ORMT apparatus. Thus, there were two pharmacological treatments: vehicle (VHC) and ketamine (KET); and two entry forms: solo (S) and dyad from the same cage and same pharmacological treatment (D). All groups had six animals. The test was performed under 60 lx. The main parameters measured in this test were the time spent exploring the objects and the time spent on the right or left side of the large box. This assay was based on other works in the literature (Ennaceur and de Souza Silva 2018).

### 2.6 Behavioral and Statistical Analyses

All recordings were analyzed manually, with the support of stopwatches and counters, by another experimenter who was blinded to the groups, sessions, or periods of the test. However, the solo/dyad variable could not be blinded. In dyad groups, the animals were individually discriminated by the sequence of entry into the apparatus. Likewise, in solo or dyad groups, each animal was analyzed separately. Using GraphPad Prism software (Version 8.0.1), normality tests were performed on all distributions, and outlier identification tests were conducted. In all multiple group analyses, Two‐way ANOVA followed by Tukey's, Sidak's, or Dunnett's multiple comparison post‐test was applied for discrimination of > 2 distributions. In 3DM analyses, a Pearson correlation was used to examine the relationship between all five parameters, and a linear regression was applied to investigate the relationship between parameters and sessions across all groups. Additionally, in the VHC‐S, VHC‐D, KET‐S, and KET‐D groups, a tracking analysis was performed using Idtracker.ai software (Romero‐Ferrero et al. 2019). In SCPP analyses, a Pearson correlation was applied between the time in the social place and the number of transitions over all test time and the last 5 min. The analyses of ORMT were limited to the first 5 min, and animals that explored the objects for less than 20 s were excluded from the analyses. The data were expressed as mean ± SEM The significant degree considered was *p* ≤ 0.05.

## 3 Results

### 3.1 General Analyses

To assess the general condition of the animals, various tests and observations were conducted. No significant differences were found between the mass of groups over any experiment (data not shown). Three animals were identified as outliers in many distributions of the 3DM test. They were excluded entirely from further analyses: one from KET‐D, one from KET‐P, and one from VHC‐P. These two P animals were from the same dyad. In general, no significant fights were observed in the cages, and there were no signs of disease, such as piloerection, weight loss, or decreased locomotion, among animals during the experiments.

### 3.2 Anxiety‐Like Behavior

#### 3.2.1 The Ketamine Induced Anxiogenic‐Like Behavior

Firstly, to assess the interaction between parameters, a Pearson correlation was performed between all five parameters, revealing significant (*p* < 0.05) and strong (*r* > 0.5 or *r* < −0.5) correlations among them (Table S1). This supports the use of these parameters for evaluating the same behavior.

Later, to assess the effect of ketamine, a two‐way ANOVA followed by Sidak's multiple comparison post hoc test was performed separately across all entry forms. Across all four entry forms in 3DM, the KET groups performed worse than the VHC groups. In solo entries, the KET‐S spent significantly less time on the arms and more time on the center in the 4th session (*p* = 0.0085; *p* = 0.0153, respectively) (Figure S2A). In D groups, the VHC treatment had better performance than the KET treatment, especially in the first sessions (Figure S2B). Similar results were observed over the C and P groups, in which the C groups expressed more differences along the test (Figure S2C,D). In general, ketamine treatment induced anxiogenic‐like behavior across all conditions of entry in the 3DM.

#### 3.2.2 The Dyads Diminished Anxiogenic‐Like Behavior Induced by Ketamine

**FIGURE 1:**
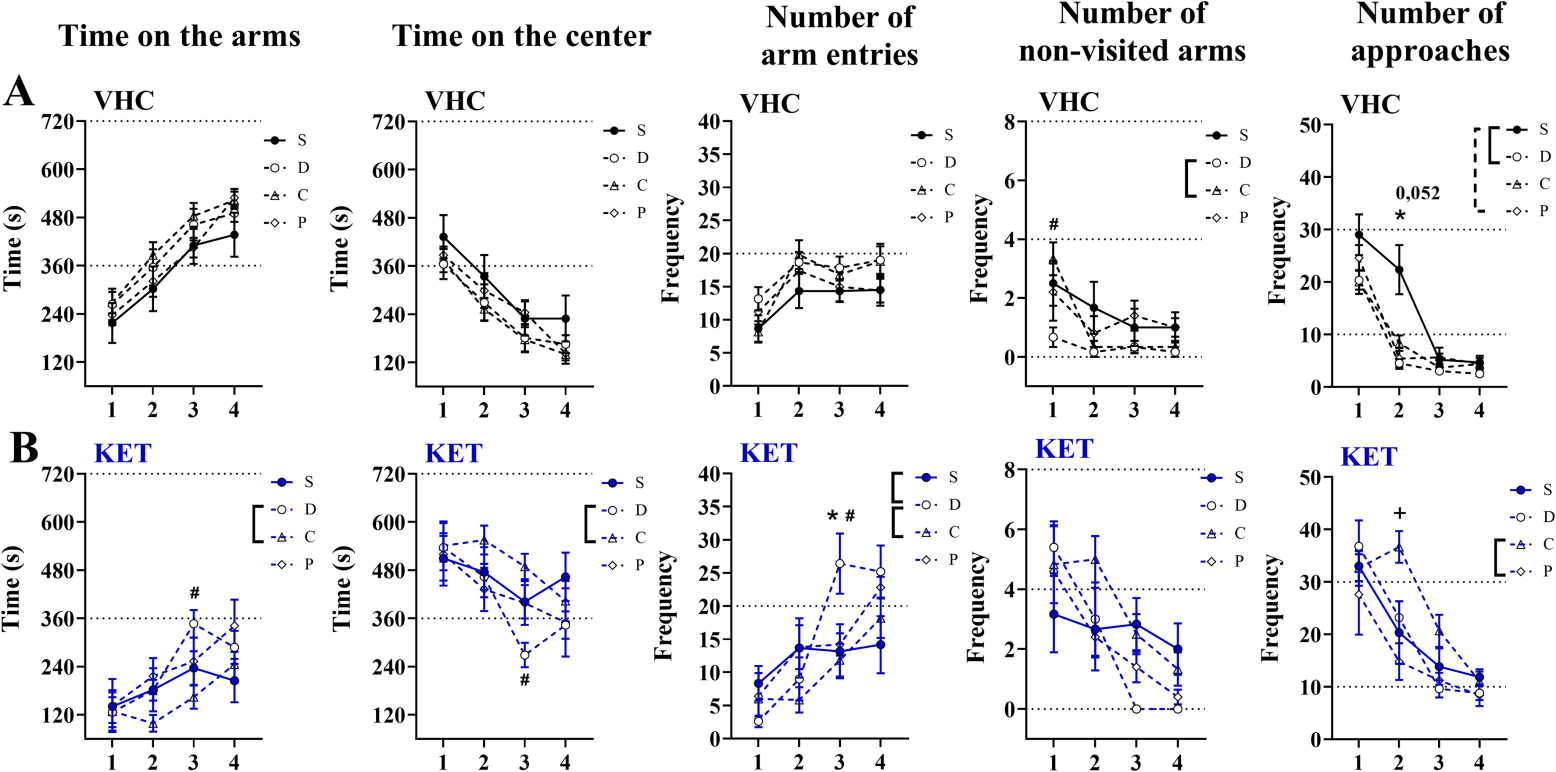
ETSB effect over anxiety‐like behavior on 3DM: Starting on the 4th day of the assay, the 3DM test was performed once a day for 4 days. The test spent 12 min by session and was performed 20 min after the administration of vehicle or ketamine (25 mg/kg). (A) ETSB effect on vehicle groups. (B) ETSB effect on ketamine groups. For all groups, the ETSB effect was analyzed by comparing the means with each other over the time on the arms, time on the center, number of arm entries, number of non‐visited arms, and the number of approaches by two‐way ANOVA followed by Tukey's multi‐comparative post‐test across four sessions. All data were expressed as mean ± SEM. *p < 0.05 between S vs. D. #*p* < 0.05 between D vs. C. +*p* < 0.05 between C vs. P. All these differences are represented on the legends by a key. The traced line on the legend of the number of approaches represents a difference almost significant between VHC‐S vs. VHC‐P (*p* = 0.052). All these signals are set above the respective sessions. *N* = 6 for VHC‐S, VHC‐D, VHCC, KET‐S and KET‐C. *N* = 5 for VHC‐P, KET‐D, and KET‐P.

To assess the social buffering effect, the groups were analyzed separately for vehicle and ketamine treatments. The analysis of time on the arms did not show any difference between VHC groups (Figure 1A). In contrast, the KET‐D group expressed a higher time on the arms in the 3rd session compared to the KET‐C group (*p* = 0.0316) (Figure 1B). A similar result was expressed over time in the center (Figure 1A,B).

The analysis of the number of arm entries also did not reveal any difference between VHC groups (Figure 1A), whereas KET‐D expressed more arm entries in the 3rd session in comparison to KET‐S and KET‐C (*p* = 0.0261; *p* = 0.0116, respectively) (Figure 1B).

The analysis of non‐visited arms revealed that the VHC‐D had significantly fewer non‐visited arms in comparison to VHC‐C in the 1st session (*p* = 0.0073) (Figure 1A). In contrast, there was no difference between KET groups (Figure 1B).

Finally, in the analysis of the number of bridge approaches, a significant decrease in VHC‐D in comparison to the VHC‐S group in the 2nd session (*p* = 0.0435), whereas in contrast between VHC‐S and VHC‐P, that decrease approached significance in the same session (*p* = 0.0517) (Figure 1A). Over the ketamine treatment, the KET‐C group expressed a higher frequency of bridge approaches in comparison to the KET‐P group in the 2nd session (*p* = 0.0056) (Figure 1B).

**FIGURE 2:**
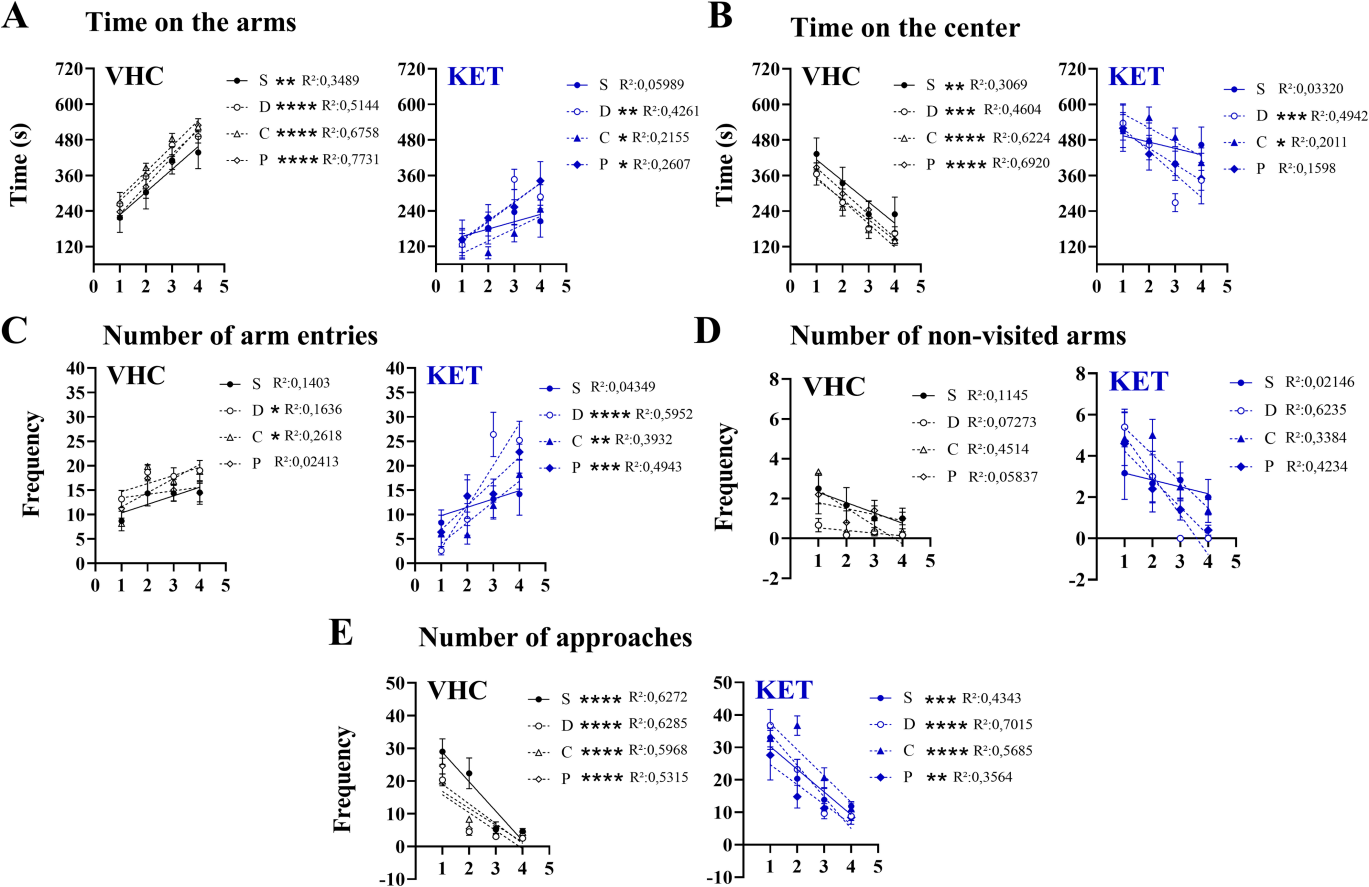
ETSB effect over habituation on 3DM: Starting on the 4th day of the assay, the 3DM test was performed once a day for 4 days. The test spent 12 min by session and was performed 20 min after the administration of vehicle or ketamine (25 mg/kg). (A) ETSB effect over time on the arms on vehicle or ketamine groups. (B) ETSB effect over time on the center on vehicle or ketamine groups. (C) ETSB effect over number of arm entries on vehicle or ketamine groups. (D) ETSB effect over number of non‐visited arms on vehicle or ketamine groups. (E) ETSB effect over number of approaches on vehicle or ketamine groups. For all groups, the ETSB effect over habituation on all vehicle or ketamine groups was analyzed by linear regression between the parameters and the sessions. All data were expressed as mean ± SEM. *p < 0.05; **p < 0.01; ***p < 0.001; ****p < 0.0001. Next to the legends are represented the, respectively, *p* and *R* ^2^ values. *N* = 6 for VHC‐S, VHC‐D, VHC‐C, KET‐S, and KET‐C. *N* = 5 for VHC‐P, KET‐D, and KET‐P.

Additionally, a linear regression was performed between each parameter and sessions to reveal the effect of sessions on each group during the test. The sessions had a significant impact over time on the arm in all groups except the KET‐S (Figure 2A). A similar result was observed on time on the center, where the effect of the session was not evident in the KET‐S and KET‐P (Figure 2B). Over the number of arm entries, the session expressed an effect on VHC‐D, VHC‐C, KET‐D, KET‐C, and KET‐P (Figure 2C). There was no effect on the number of non‐visited arms (Figure 2D) and a significant impact across all groups on the number of bridge approaches (Figure 2E). This suggests that ketamine impaired habituation and that it was mitigated by dyad groups. Moreover, the tracking analysis revealed a preference for the proximal area of bridges in all groups, especially in the 1st session. In contrast, during the 4th session, the exploration was wider; however, the KET‐S group continued to express a strong preference for the proximal area of the bridges (Figure S3).

In summary, the tests indicated a better performance of VHC‐D (i.e., weak anxiolytic‐like effect). The same was observed robustly in KET‐D over KET‐S and KET‐C (i.e., ETSB), as well as a significantly worse performance of KET‐S across sessions, as demonstrated by linear regression and tracking analysis (i.e., impairment of habituation). In addition, KET‐P did not show as strong a performance as KET‐D; however, future works must corroborate a significant difference between familiar dyads from different treatments.

#### 3.2.3 The Familiarity Increased the Anxiolytic‐Like Effect of Dyad Groups

**FIGURE 3:**
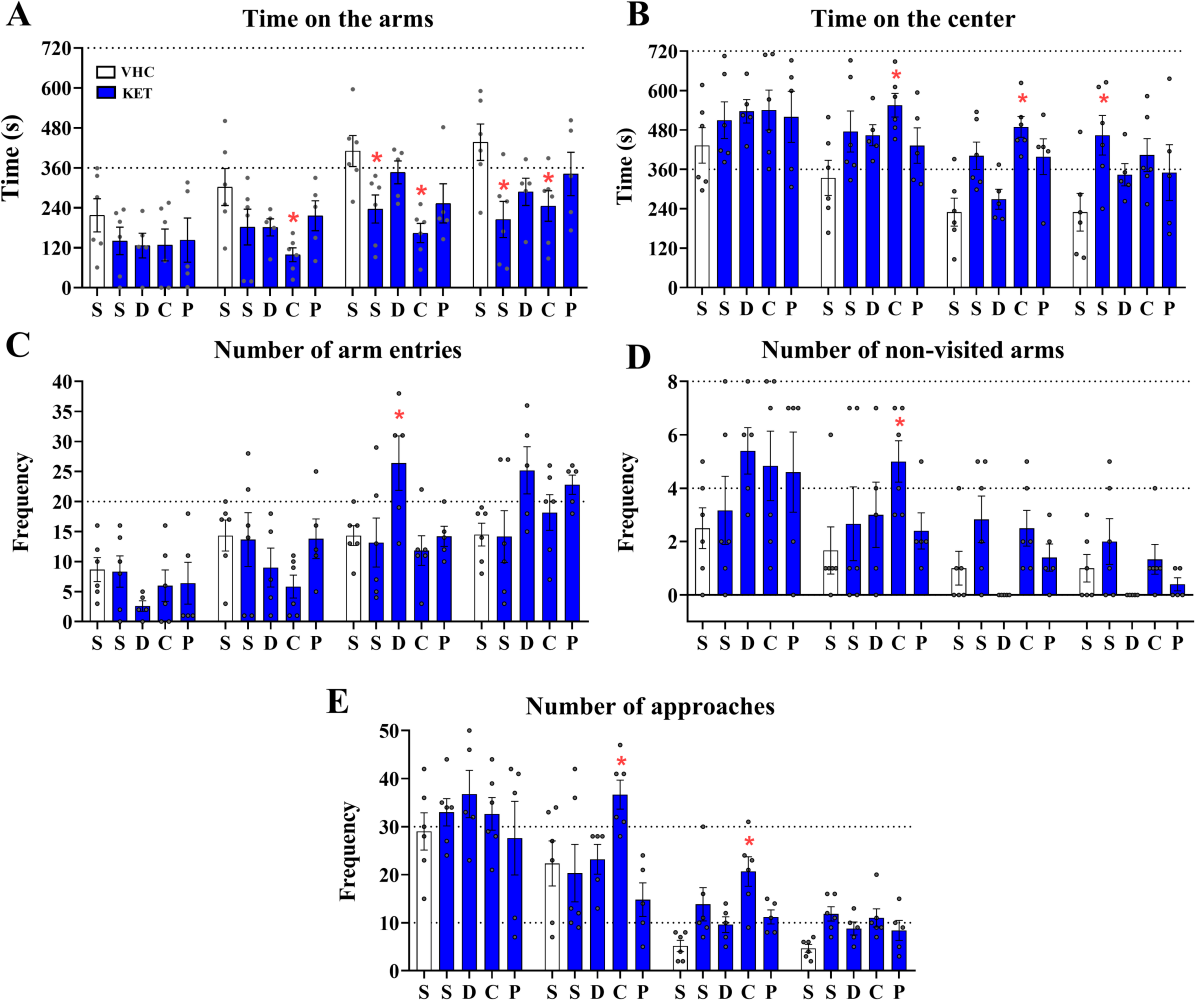
Familiarity effect on 3DM: Starting on the 4th day of the assay, the 3DM test was performed daily for four consecutive days. The test lasted 12 min per session and was performed 20 min after the administration of vehicle or ketamine (25 mg/kg). (A) Familiarity effect over time on the arms of the ketamine groups. (B) Familiarity effect over time on the center of ketamine groups. (C) Familiarity effect on the number of arm entries in the ketamine groups. (D) Familiarity effect over the number of non‐visited arms in the ketamine groups. (E) The familiarity effect on the number of approaches in the ketamine groups. White bars represent the VHC‐S and blue bars represent the ketamine groups. For all groups, the familiarity effect was analyzed by comparing the means of ketamine groups against the VHC‐S mean over the time on the arms, time on the center, number of arm entries, number of non‐visited arms, and the number of approaches by two‐way ANOVA followed by Dunnett's multiple comparison post‐test. All data were expressed as mean ± SEM. *p < 0.05 against VHC‐S. All * are set above the respective groups. *N* = 6 for VHC‐S, KET‐S, and KET‐C. *N* = 5 for KET‐D and KET‐P.

To better determine the performance of KET groups, they were compared to VCL‐S performance over all five parameters. Over time on the arms, the KET‐C spent less time in the 2nd, 3rd, and the 4th session, whereas the KET‐S spent less time in the 3rd and 4th session (*p* < 0.05 for all) (Figure 3A).

Over time on the center the KET‐C spent more time in the 2nd and in the 3rd session, whereas the KET‐S spent more time in the 4th session (*p* < 0.05 for all) (Figure 3B).

The number of arm entries analysis revealed that KET‐D had a significantly higher frequency in the 3rd session (*p* = 0.022) (Figure 3C). Differently, the number of non‐visited arms revealed a higher frequency of non‐visited arms of KET‐C in the 2nd session (*p* = 0.0264) (Figure 3D). Finally, over the number of bridge approaches analysis, the KET‐C showed a higher frequency in the 2nd and in the 3rd session (*p* = 0.0121; *p* = 0.0058, respectively) (Figure 3E).

In summary, the analyses revealed better performance for KET‐D and worse performance for KET‐C. Despite the great differences between the VHC‐S and KET‐C, the linear regression analysis and the analysis of the 4th session revealed worse performance of KET‐S than KET‐C (i.e., impairment of habituation). Therefore, the anxiolytic‐like effect of dyad groups was higher in dyads from the same cage (KET‐D and KET‐P). Despite the worst performance of KET‐C in the first three sessions, that group expressed more dependence on sessions and fewer differences with VHC‐S in the 4th session. Thus, there was a weak and slow improvement in habituation in dyads of different cages (KET‐C) than in KET‐S.

#### 3.2.4 The Performance of KET‐C Is Not Dependent on Familiarity

Similar to the above 3DM test, all five parameters were significantly (*p* < 0.05) and strongly (*r* > 0.5 or *r* < −0.5) correlated positively or negatively by Pearson correlation analysis at this test (Table S2).

Moreover, the above results (Figure 3) indicated that familiarity increased the anxiolytic‐like effect in the KET groups, and a weak and slow anxiolytic‐like effect was observed in the KET‐C group. Therefore, this effect on KET‐C could be dependent on the gradual increase in familiarity of KET‐C dyads along the test. Thus, to address this issue, a 5‐day experiment with 10 min of test daily (2.3.3.) was performed.

The analyses of SC and DC revealed few differences between them: There were no significant differences over time on the arms, time on the center, number of arm entries, or number of non‐visited arms across the five sessions. The only significant difference points to a weak better performance of DC in the 4th session of the test: DC expresses significantly less frequency of bridge approach than SC (*p* = 0.043); however, that difference was not kept in the 5th session (*p* = 0.8607) (Figure S4). In addition, the linear regression analysis revealed that both groups showed a strong dependence on the sessions across the test in all five parameters analyzed (habituation) (Figure S5).

In summary, under this condition, no evidence indicating that SC (i.e., more familiarity) expresses less anxiety‐like behavior in comparison to DC (i.e., less familiarity) is found. Therefore, those results suggest that the weak and slow anxiolytic‐like effect in the previous KET‐C (habituation) was induced by the entry form (dyad) and not by the increase in familiarity along the test.

### 3.3 Social Reward

**FIGURE 4:**
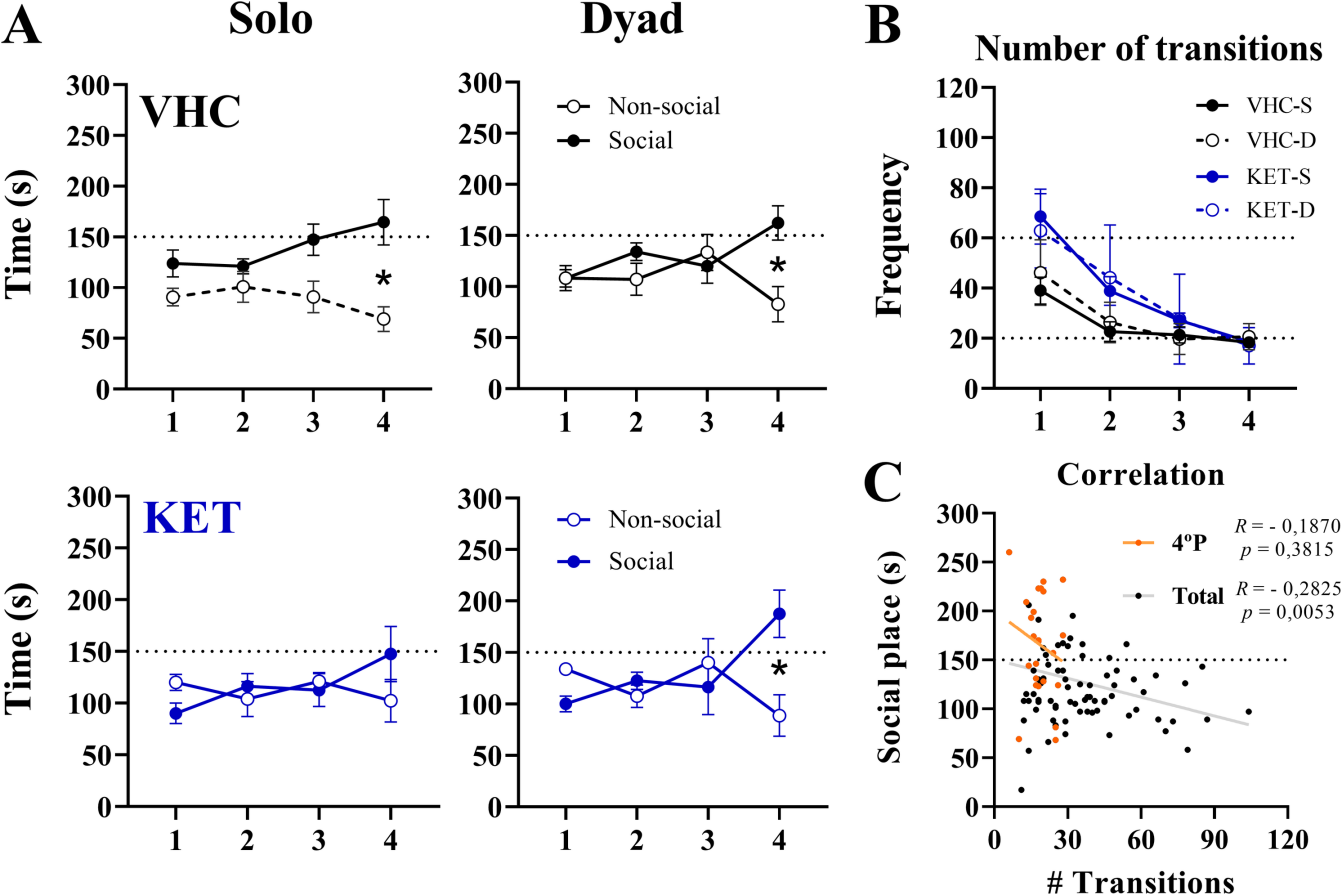
Dyad effect on social reward: After 24 h of social isolation, on the 4th day of the assay, the SCPP test was performed. The test spent 20 min and was performed 20 min after the administration of vehicle or ketamine (25 mg/kg). (A) Dyad effect over place preference in each period of the test. (B) Number of transitions. (C) Pearson correlation between the time on the social place and the number of transitions over the total time or over the 4th period; the respective *p* and *R* values are represented next to the legends. For all groups, the dyad effect was analyzed by comparing the means with each other across time on social place, time on non‐social place, and number of transitions using a two‐way ANOVA followed by Sidak's multiple comparison post‐hoc test. All data were expressed as mean ± SEM. *p < 0.05 against social vs. non‐social. The * are set above the respective periods. *N* = 6 for all groups.

The above results revealed better performance for KET‐D and VHC‐D; therefore, these groups were chosen for further experiments. Hence, the SCPP analyses revealed that VHC‐S (*p* = 0.024), VHC‐D (*p* = 0.0316), and KET‐D (*p* = 0.0361) had a significant preference for social place in the 4th period of the test, whereas the KET‐S group did not (*p* = 0.6136) (Figure 4A). In addition, we did not find any significant difference in the number of transitions between groups in each period of the test (Figure 4B). Finally, through Pearson correlation, we found a significant (*p* = 0.0053) negative correlation between the number of transitions and the time in the social place across the total periods of the test (*R* = 0.2825). However, there was no significant correlation (*p* = 0.3815) in the 4th period of test (where there is a significant difference between groups) between the number of transitions and the time in the social place (*R* = 0.187) (Figure 4C).

In summary, the analyses revealed an impairment of social reward induced by ketamine that was restored by the presence of another conspecific animal from the same cage (KET‐D). This effect was not dependent on a decrease in transitions across the test.

### 3.4 Episodic Memory

**FIGURE 5:**
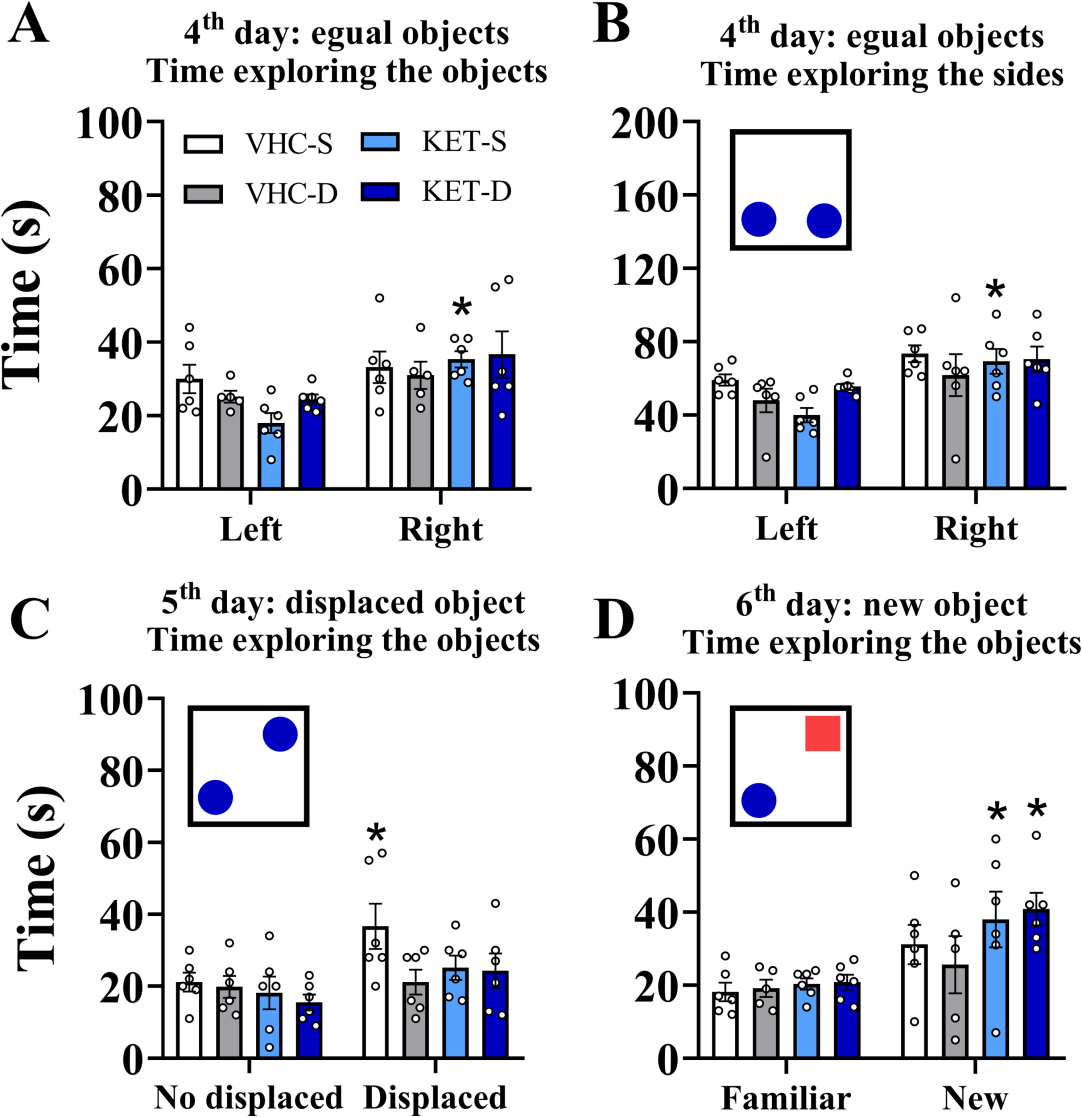
Dyad effect on episodic memory: Since the 4th day of the assay, the ORMT test was performed. The test spent 10 min and was performed 20 min after the administration of vehicle or ketamine (25 mg/kg). On the 4th day, the animals were exposed to two equal objects; on the 5th day, one object was displaced; and on the 6th day, the displaced object was replaced with a new one. (A) Dyad effect on the 4th day, over time, exploring the objects. (B) Dyad effect on the 4th day over time, exploring the sides. (C) Dyad effect on the 5th day (LRM) over the time exploring the objects. (D) Dyad effect on the 6th day (ORM) over the time exploring the objects. For all groups, the dyad effect was analyzed by comparing the means of groups with each other, as well as comparing left vs. right, no displaced vs. displaced, and familiar vs. new within each group over time. This analysis explored the objects and time spent exploring the sides using a two‐way ANOVA, followed by Sidak's multiple comparison post‐test. All data were expressed as mean ± SEM. *p < 0.05 against left, no displaced or familiar mean of the respective group. The * are set above the respective group. *N* = 6 for all groups.

The analysis of the 4th day of the test did not reveal any significant difference between groups over the time exploring the objects (*p* > 0.05 for all). However, the KET‐S expressed a significant preference for the right object (*p* = 0.0074) (Figure 5A). Additionally, on this day, the KET‐S was the only group to express preference for the right side of the large box (*p* = 0.0029), with no significant difference between groups on either the left or right side (*p* > 0.05 for all) (Figure 5B). Thus, this object preference on the 4th day of the test by KET‐S was attributed to side preference induced by spatial cues.

Moreover, on the 5th day of the test, just the VHC‐S expressed preference in time exploring objects for displaced object (*p* = 0.0169) (Figure 5C). Differently, on the 6th day, just the KET‐S and KET‐D expressed significant preference for a new object (*p* = 0.0373; *p* = 0.0161, respectively) (Figure 5D).

Finally, there was no significant difference (*p* > 0.05) between groups concerning the left or right side, the left or right object, the displaced or non‐displaced object, or the familiar or new object.

In summary, KET‐S expresses a natural preference for the right side and new object, without preference for the displaced object, whereas VHC‐S expresses a preference for the displaced object only. Thus, ketamine treatment induced a spatial memory deficit and an improvement in familiarity memory. Additionally, the presence of another conspecific animal impaired spatial memory compared to the control group and had no effect in the ketamine‐treated group.

## 4 Discussion

To our knowledge, this was the first work to demonstrate the anxiogenic‐like effect of ketamine in 3DM. This anxiogenic‐like effect induced by ketamine is also observed in current tests of unconditioned anxiety (Acevedo et al. 2023; Akillioglu et al. 2012; Amorim et al. 2018). Similarly, this effect was demonstrated with MK801, another allosteric antagonist of N‐methyl‐D‐aspartate (NMDA) receptor, in 3DM (Abuhamdah et al. 2016; A. Ennaceur et al. 2011) or in current tests of unconditioned anxiety (Lee and Zhou 2019). Likewise, SCZ patients have a higher prevalence of anxiety disorders (Achim et al. 2011). Thus, these results contribute to the face validity of the SCZ model induced by NMDA receptor antagonists. The tracking analysis also revealed a characteristic behavior of exploring the proximal areas of bridges, which is lost along the sessions according to a decrease in anxiety status (i.e., habituation) through a wider exploration of the distal areas of 3DM. A similar effect was recently observed in a previous study using another tracking software (Monteiro et al. 2025).

Additionally, in this work, all groups analyzed in 3DM expressed a great or weak anxiolytic‐like effect throughout the sessions. This effect is a characteristic of 3DM (Abuhamdah et al. 2015; Abuhamdah et al. 2016; Ennaceur et al. 2006; Ennaceur et al. 2008; Ennaceur et al. 2011), whose paradigms improve the assessment of anxiety‐like behavior in 3DM and which is not found in current tests of unconditioned anxiety (e.g., elevated plus maze, open field, light/dark box) (Ennaceur and Chazot 2016). In addition, correlation analysis suggests that all analyzed parameters belong to the same behavioral domain. Therefore, the animals systematically learned, over the course of the sessions, that the distal areas of 3DM are not dangerous. Thus, beyond the assessment of the status of anxiety, the 3DM tests assess a learning curve related to anxiety (i.e., habituation). Moreover, this decrease in fear/anxiety occurred naturally, not through conditioning, and it is not a behavioral change related to a task previously learned, so it cannot be called Extinction (Dunsmoor et al. 2015; Monteiro et al. 2025). In addition, habituation is a paradigm applied to decrease the anxiety status in many behavioral test protocols, where animals are previously presented to the apparatus or experimental room before the test.

In this way, it is correct to affirm over anxiety‐like behavior assessing that: ketamine impaired habituation; the ETSB improved the habituation in groups treated with ketamine; and that higher familiarity induces a faster habituation. On the other hand, despite the extensive literature supporting the relief effect of social buffering in naïve animals (Kiyokawa 2018; Kiyokawa and Hennessy 2018), in this work, we did not find a great effect of ETSB over vehicle groups in 3DM. However, this result could be due to the fact that C57 mice are a “low‐anxiety” strain (Ennaceur et al. 2006). Thus, a floor effect may have confounded the analysis, preventing the ETSB from decreasing the anxiety status along the test in vehicle groups. Additionally, the manipulations before the test could also have reduced the anxiety status of mice (Abuhamdah et al. 2015). That floor effect is also present in the ketamine groups, where the KET‐D group reached a low level of anxiety/fear status in the 3rd session. In contrast, the other ketamine groups reached that status in the 4th session. Additionally, our results support that the relief effects of social buffering could be applied in mouse models of psychiatric disorders. Therefore, in this work, the ETSB improved the habituation in groups whose anxiety status was increased by ketamine. Similarly, Mikami et al. (2016, 2020) reported that ETSB improved the extinction of fear induced by conditioning in rats, and Klein et al. (2015) reported ETSB in a model of stress induced new environment in mice through evaluating cell response in the paraventricular nucleus of the hypothalamus. Thus, it supports the notion that the presence of a conspecific animal accelerates learning related to an emotional fear status conditioned or induced by a drug. This broad spectrum of contexts modulated by social buffering encourages the investigation of its effects in other models.

Furthermore, the nature of conspecific animals modulated the ETSB effect. In this work, dyads from the same cage and the same pharmacological treatment (i.e., D groups) had a faster habituation. Similarly, the presence of a familiar conspecific was more effective to relieve behavioral and corticosterone stress response induced by new environment than unfamiliar in periadolescent rats (Terranova et al. 1999), as well as that same effect of relief was also observed in conditioned fear through behavioral and cell response in the paraventricular nucleus of the hypothalamus and in the amygdala in adult rats (Kiyokawa et al. 2014). Therefore, the magnitude of social buffering is modulated by social interaction before and during stress, in which higher familiarity promotes greater social buffering. On the other hand, dyads from the same cage and different pharmacological treatments (i.e., P groups) did not show an excellent performance related to anxiety‐like behavior. However, related to episodic‐like memory and in a model of autism spectrum disorder (BTBR strain), BTBR mice showed an improvement in episodic‐like memory when performing a memory test with C57 mice compared to when performing with other BTBR mice (Lipina and Roder 2013). That supports the investigation of a wide range of domains related to conspecific nature in psychiatric mouse models.

Related to the performance of KET‐C groups, no improvement in habituation associated with higher familiarity was found in the SC than in the DC group; however, the time together of the SC dyad during the test was not sufficient to promote the social buffering effect observed in KET‐D. Previous work has demonstrated that this period of time together in mice should be extended beyond 3 weeks to promote empathy behavior significantly better than mice from different cages (Langford et al. 2006).

Beyond the social buffering effect in alleviating stress, our results suggest that the presence of a familiar conspecific restores the social reward, which was impaired by ketamine. In addition, despite ketamine inducing an increase in exploration (Lai et al. 2014), there was no significant difference between groups concerning the frequency of transitions. Correlation analysis supports that, in the 4th session, the frequency of transition did not affect the time in the social place. Social deficits are a common feature in a wide range of psychiatric disorders (American Psychiatric Association and American Psychiatric Association 2013), as well as a great range of psychosocial interventions that involve interventions in the social environment in SCZ (Keepers et al. 2020). In this way, even in an enriched environment, works using SCZ mouse models, social behaviors are ignored or assessed as a symptom (Bator et al. 2018; Burrows et al. 2015; Faatehi et al. 2019; Harb et al. 2021; Huang et al. 2021; Koseki et al. 2012; McOmish et al. 2008; Murueta‐Goyena et al. 2018; Zhu and Grace 2021). However, the social environment emerges as a target for exploration due to its importance and potential to modulate negative and cognitive symptoms in SCZ. Recent studies using enriched environment protocols, where the social stimulus is increased, have demonstrated promising outcomes in SCZ mouse models (Aykan et al. 2024; Huang et al. 2021). Likewise, this must be expanded to mouse models of other psychiatric disorders.

Regarding the episodic memory assessed by ORMT, in the same conditions that promote anxiogenic‐like behavior, weak habituation, and deficits in social reward, ketamine impaired LRM but not ORM. Additionally, the presence of another conspecific animal did not promote any improvement in ORM or LRM over vehicle or ketamine treatment. In SCZ patients, episodic memory is impaired; however, recollection (which involves spatial memory recognition) is more affected than familiarity (Libby et al. 2013). On the other hand, over a wide range of protocols using ketamine, most studies assessing the ORM rather than the LRM, among them, most report a deficit in ORM; however, other studies report no effect or even an improvement in the impact on ORM induced by ketamine (Pitsikas 2018). In contrast, social isolation induced a clear deficit in recognition memory (ORM and LRM), which could be restored by an enriched environment (Shang et al. 2024). However, few works demonstrated the effect of a conspecific animal during the task: de Franca Malheiros et al. (2020) reported an improvement in episodic memory in rats (de Franca Malheiros et al. 2020), whereas Lipina and Roder (2013) reported an improvement in LRM in C57 mice and in BTBR mice (Lipina and Roder 2013). Moreover, mice prefer to explore familiar objects that have been previously conditioned to the presence of another conspecific animal (Ross et al. 2024), and C57 mice exhibit high levels of prosocial behavior (Sankoorikal et al. 2006). This preference for social context in C57 mice supports the SCPP test (Panksepp and Lahvis 2007). Therefore, few data are available to help us discriminate between reporting that the presence of another conspecific animal impaired ORM, which is not supported by few works in the literature, or reporting that our protocol was unable to reveal the effect of the presence of another conspecific animal on episodic memory, concerning the differences of protocols (e.g., four objects and one chamber in previous works and two objects and two chambers at this work) and the high preference for social cues in C57 mice (distraction effect). Hence, we suggest that new protocols assessing progressive learning will be able to answer that issue.

Nevertheless, in 3DM, the KET‐S showed a weak and non‐significant increase in the number of arm entries and a decrease in non‐visited arms across the sessions. In contrast, the KET‐D significantly increased the number of arm entries across the sessions, and all animals reached 0 non‐visited arms by the 3rd session. Similarly, the VHC‐D expressed values of non‐visited arms below 1 since the 1st session. Therefore, whereas the KET‐S continues to repeat the arm visited, even reaching a mean number of arm entries above 14 in the 4th session, the KET‐D and VHC‐D perform a more efficient exploration by visiting more different arms. Hence, unlike ORMT analysis, these data in 3DM suggest that the presence of another conspecific animal improves cognition related to spatial navigation along the test, which supports a distraction effect in ORMT.

Some limitations at this work were observed: despite the behavioral outcomes, analyses of corticosterone and brain areas related to stress, anxiety, social reward, and episodic memory would support the identification of responsible pathways of social cognition; similarly, despite our results pointing to social buffering, the presence of a conspecific animal could also promote social transference of pain, fear or stress (Walsh et al. 2023). Therefore, the modulation of the social context demands a sharp analysis of confounding results. In addition, despite the presence of another conspecific animal promoting an anxiolytic‐like effect, a faster habituation, and restoring the preference for social cues, in ORMT, this presence may have promoted distraction, which impairs the analysis and suggests the creation of new tests to assess episodic memory performance in dyads or groups. Moreover, although male sex is more affected by SCZ (Solmi et al. 2023), this work was limited to using only male mice. Likewise, the issue of familiarity and its effect on prosocial behaviors and memory must be explored further in the future. Finally, the reliability of the data may be impacted by the small sample size (*N*); however, this concern is mitigated by the large effect size observed between the distributions (data not shown).

## 5 Conclusion

In summary, the presence of another conspecific animal improves cognition related to anxiety, spatial navigation, and social behaviors in the SCZ model induced by ketamine. Within the clinical scope, social interventions are a crucial tool to treat SCZ (Keepers et al. 2020). On the other hand, the modulation of the social environment raises as a potential field to be explored within the preclinical scope, aiming at the study of the physiology of social cognition or the emergence of new treatments for mental disorders. Particularly in SCZ, our findings support the use of non‐pharmacological interventions to treat negative and cognitive symptoms.

## Author Contributions

**Rayan Fidel Martins Monteiro:** conceptualization, formal analysis, investigation, methodology, project administration, visualization, writing – original draft. **Marcos Vinícius Lebrego Nascimento:** investigation. **Klinsmann Thiago Lima:** investigation, methodology, writing – review and editing. **Valdina Solimar Lopes Cardoso:** methodology. **José Ramon Gama Almeida:** investigation. **Wellington Junior Taisho Nagahama Costa:** investigation. **Bruno Eduardo Godinho Teixeira:** investigation. **Vinicius Teles Shirakura:** investigation. **Soraya Amin Souza:** investigation. **Juliana Silva Cassoli:** conceptualization, methodology, resources, supervision, writing – review and editing. **Gilmara de Nazareth Tavares Bastos:** conceptualization, methodology, resources, supervision, writing – review and editing.

## Funding

This work was supported by the National Council of Scientific and Technological Development (CNPq) and by the Coordenação de Aperfeiçoamento de Pessoal de Nível Superior (CAPES).

## Conflicts of Interest

The authors declare no conflicts of interest.

## Supporting information


**Figure S1:** Experimental designs: (A) 3DM and experimental groups. (B) experimental design to assessing anxiety‐like behavior. (C) experimental design to discriminate KET‐C groups. (D) SCPP apparatus. (E) experimental design to assessing social reward. (F) ORMT apparatus. (G) experimental design for assessing episodic memory.

**Figure S2:** Ketamine effect on 3DM: Starting on the 4th day of the assay, the 3DM test was performed daily for four consecutive days. The test spent 12 min per session and was performed 20 min after the administration of vehicle or ketamine (25 mg/kg). (A) Ketamine effect on S groups. (B) Ketamine effect on D groups. (C) Ketamine effect on C groups. (D) Ketamine effect on P groups. For all groups, the ketamine effect was analyzed against the vehicle groups over time on the arms, time on the center, number of arm entries, number of non‐visited arms, and number of approaches using a two‐way ANOVA followed by Sidak's multiple comparison post‐test. All data were expressed as mean ± SEM *p < 0.05. *N* = 6 for VHCS, VHC‐D, VHC‐C, KET‐S, and KET‐C. *N* = 5 for VHC‐P, KET‐D, and KET‐P.

**Figure S3:** Behavioral characterization of habituation on 3DM. Starting on the 4th day of the experiment the 3DM test was performed once a day for 4 days. The test spent 12 min by session and was performed 20 min after the administration of vehicle or ketamine (25 mg/kg). Tracking analysis by Idtracker.ai software of VHC‐S, VHC‐D, KET‐S, KET‐D on 1st (D1) and on the 4th (D4) day of the day.

**Figure S4:** Anxiety‐like behavior in groups of different cages treated with ketamine: Starting on the 4th day of the experiment, the 3DM test was performed once a day for 5 days. The test spent 10 min per session and was performed 20 min after the administration of ketamine (25 mg/kg). Same dyad along the test (SC), different dyad along the test (DC). For all groups, anxiety‐like behavior was analyzed by comparing the means with each other over time on the arms, time on the center, number of arm entries, number of non‐visited arms, and number of approaches using a two‐way ANOVA followed by Sidak's multiple comparison post hoc test. All data were expressed as mean ± SEM *p < 0.05 between SC vs. DC. The * is set above the respective sessions. *N* = 6 for all groups.

**Figure S5:** Habituation in groups of different cages treated with ketamine: Starting on the 4th day of the assay, the 3DM test was performed once daily for 5 days. The test spent 10 min per session and was performed 20 min after the administration of ketamine (25 mg/kg). (A) habituation over time on the arms of KET‐C groups. (B) Habituation over time on the center of KET‐C groups. (C) Habituation over the number of arm entries on KET‐C groups. (D) Habituation over the number of non‐visited arms on KET‐C groups. (E) Habituation over the number of approaches on KET‐C groups. Same dyad along the test (SC), different dyad along the test (DC). For all groups, habituation in the KET‐C groups was analyzed using linear regression between the parameters and the sessions. All data were expressed as mean ± SEM **p < 0.01; ***p < 0.001; ****p < 0.0001. Next to the legends are the respective *p* and *R* ^2^ values. *N* = 6 for all groups.

**Table S1:** Correlation between 3DM parameters.

**Table S2:** Correlation between 3DM parameters of KET‐C groups.

## Data Availability

The data that support the findings of this study are openly available in Figshare at 10.6084/m9.figshare.30675206. This includes raw behavioral data for the 3D maze, social conditioned place preference, and object recognition memory tests.
