# Supplementary figures and images for: Conspecific Presence Promotes Social Buffering, Restores Social Reward, and Enhances Spatial Navigation in a Ketamine‐Induced Model of Schizophrenia in Mice
Source: Eur J Neurosci. 2025 Dec 17;62(12):e70359. doi: 10.1111/ejn.70359 (PMC12710120; doi:10.1111/ejn.70359)

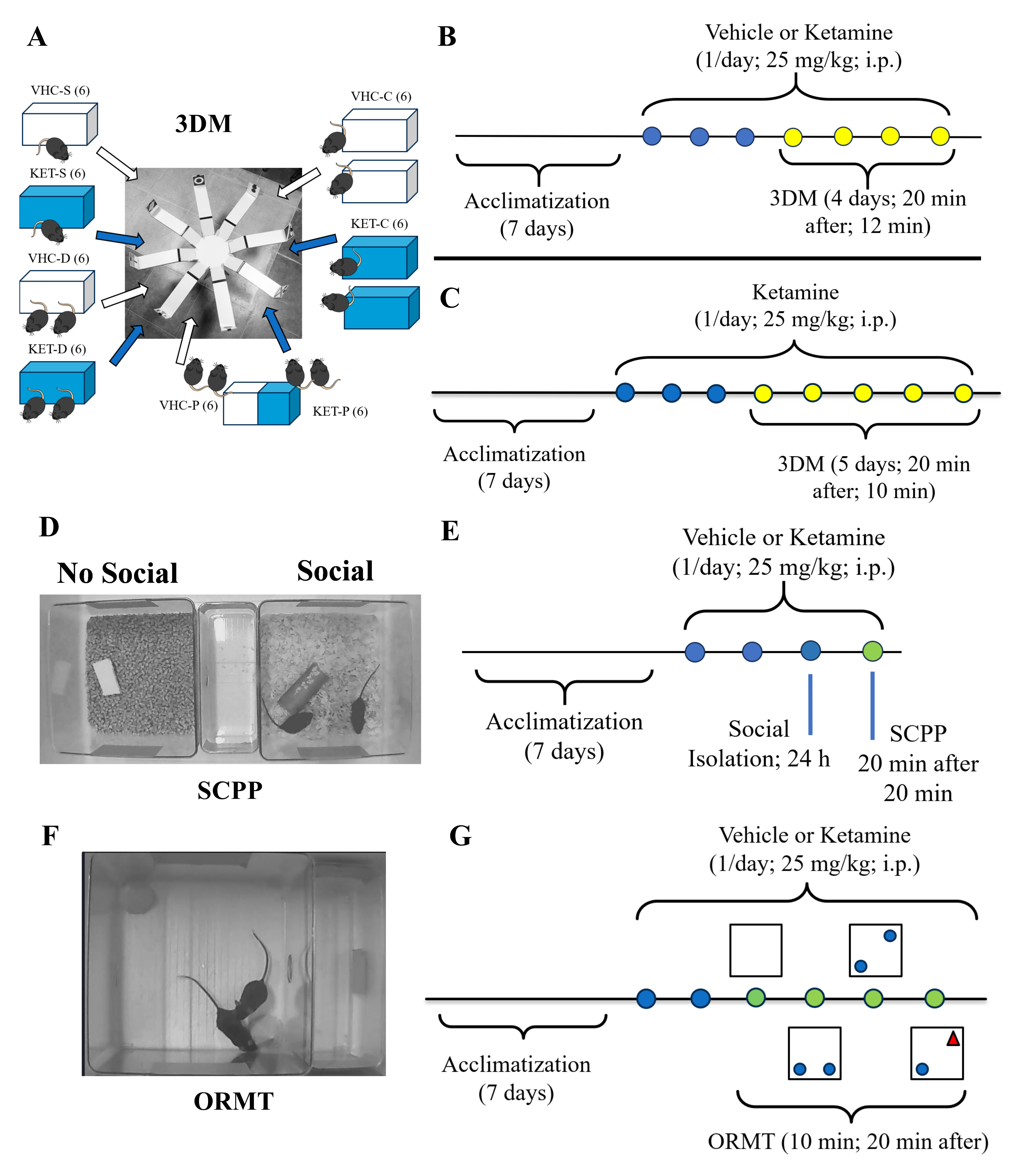

Supplement: Supplementary file 1 — Figure S1: Experimental designs: (A) 3DM and experimental groups. (B) experimental design to assessing anxiety‐like behavior. (C) experimental design to discriminate KET‐C groups. (D) SCPP apparatus. (E) experimental design to assessing social reward. (F) ORMT apparatus. (G) experimental design for assessing episodic memory. [file EJN-62-0-s005.tif]

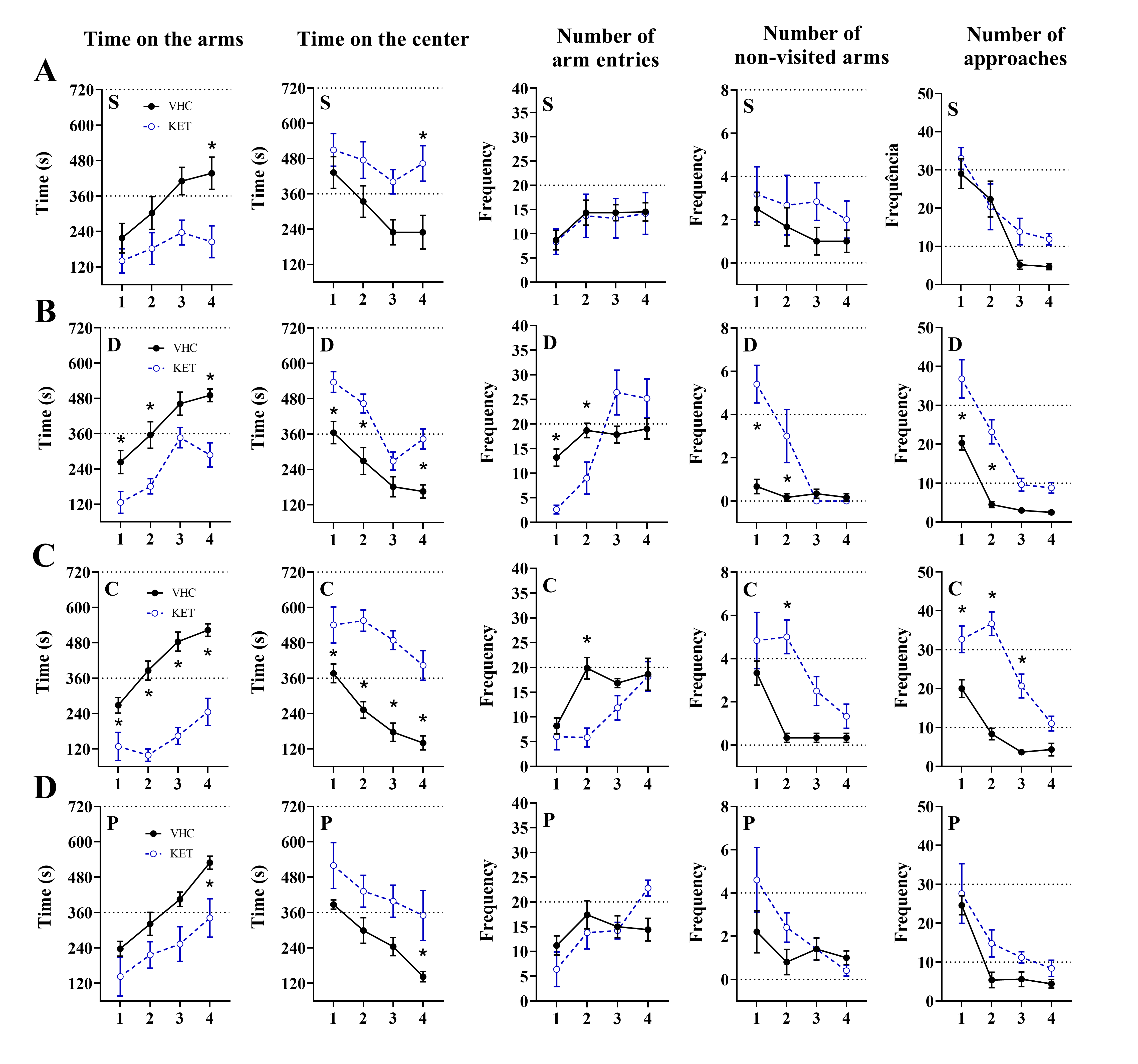

Supplement: Supplementary file 2 — Figure S2: Ketamine effect on 3DM: Starting on the 4th day of the assay, the 3DM test was performed daily for four consecutive days. The test spent 12 min per session and was performed 20 min after the administration of vehicle or ketamine (25 mg/kg). (A) Ketamine effect on S groups. (B) Ketamine effect on D groups. (C) Ketamine effect on C groups. (D) Ketamine effect on P groups. For all groups, the ketamine effect was analyzed against the vehicle groups over time on the arms, time on the center, number of arm entries, number of non‐visited arms, and number of approaches using a two‐way ANOVA followed by Sidak's multiple comparison post‐test. All data were expressed as mean ± SEM *p < 0.05. N = 6 for VHCS, VHC‐D, VHC‐C, KET‐S, and KET‐C. N = 5 for VHC‐P, KET‐D, and KET‐P. [file EJN-62-0-s004.tif]

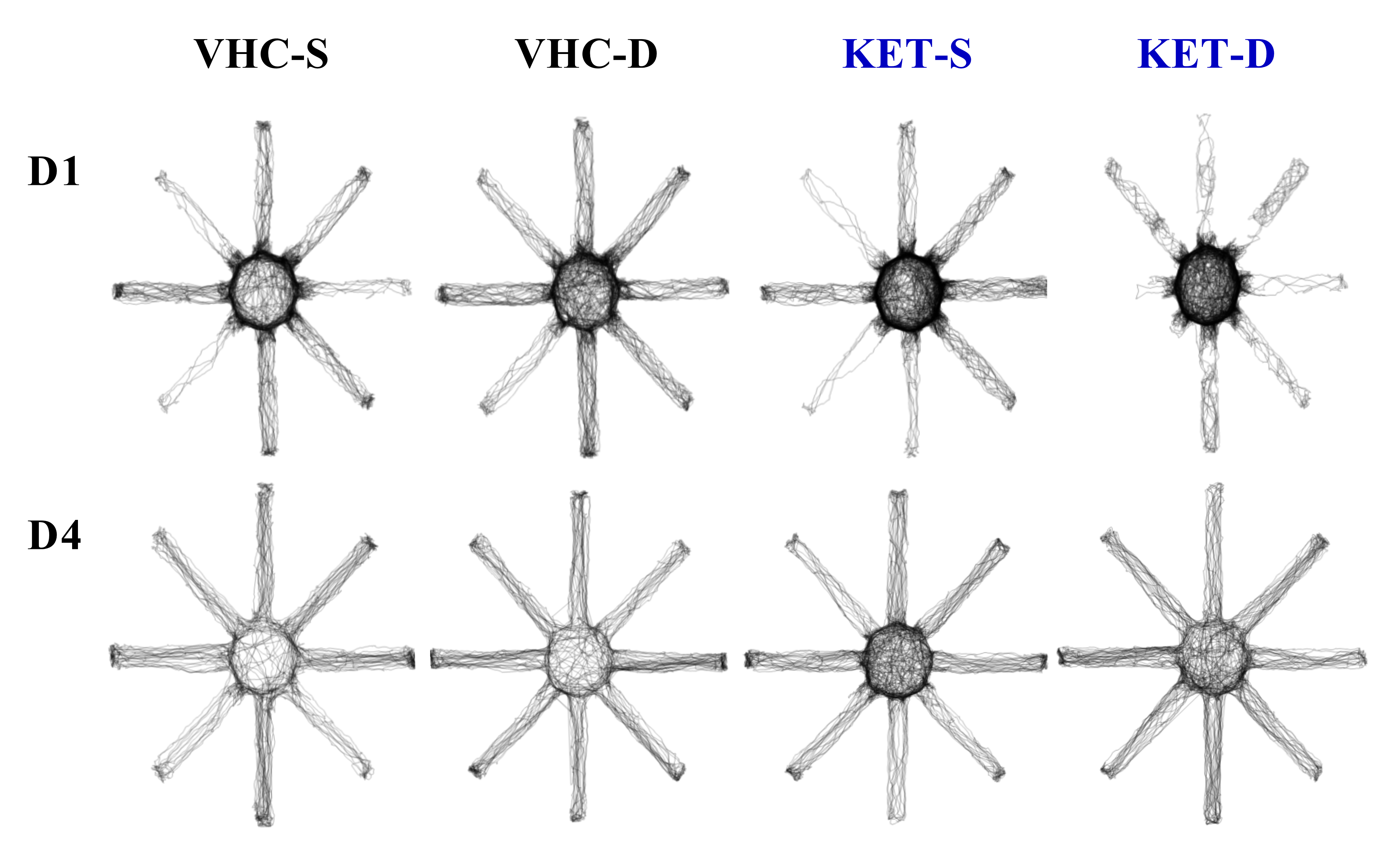

Supplement: Supplementary file 3 — Figure S3: Behavioral characterization of habituation on 3DM. Starting on the 4th day of the experiment the 3DM test was performed once a day for 4 days. The test spent 12 min by session and was performed 20 min after the administration of vehicle or ketamine (25 mg/kg). Tracking analysis by Idtracker.ai software of VHC‐S, VHC‐D, KET‐S, KET‐D on 1st (D1) and on the 4th (D4) day of the day. [file EJN-62-0-s007.tif]

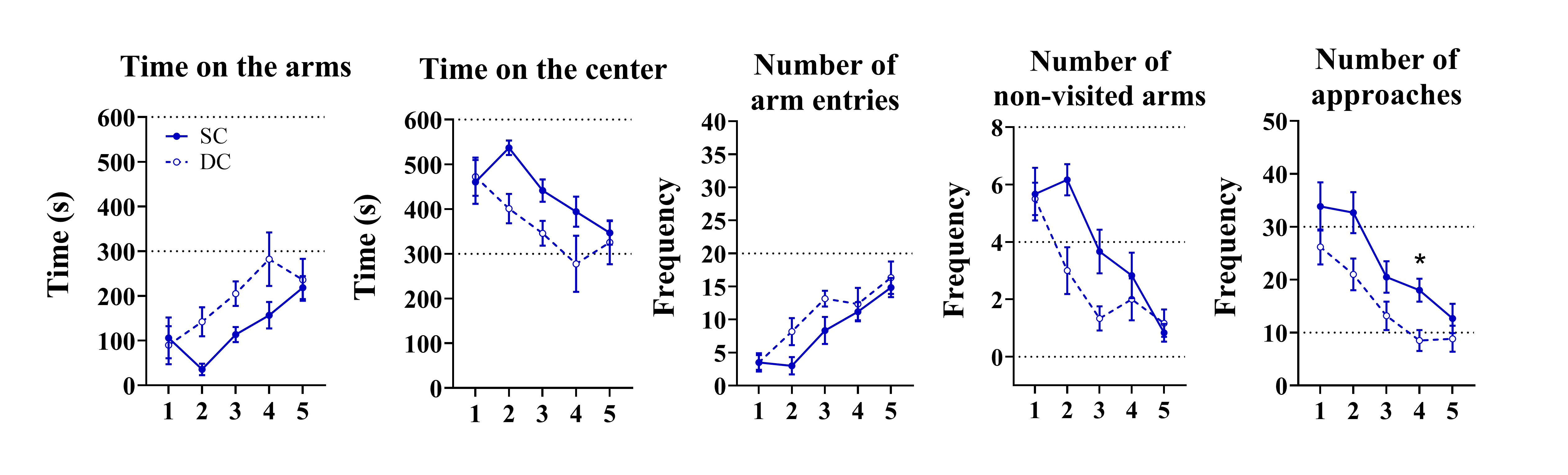

Supplement: Supplementary file 4 — Figure S4: Anxiety‐like behavior in groups of different cages treated with ketamine: Starting on the 4th day of the experiment, the 3DM test was performed once a day for 5 days. The test spent 10 min per session and was performed 20 min after the administration of ketamine (25 mg/kg). Same dyad along the test (SC), different dyad along the test (DC). For all groups, anxiety‐like behavior was analyzed by comparing the means with each other over time on the arms, time on the center, number of arm entries, number of non‐visited arms, and number of approaches using a two‐way ANOVA followed by Sidak's multiple comparison post hoc test. All data were expressed as mean ± SEM *p < 0.05 between SC vs. DC. The * is set above the respective sessions. N = 6 for all groups. [file EJN-62-0-s001.tif]

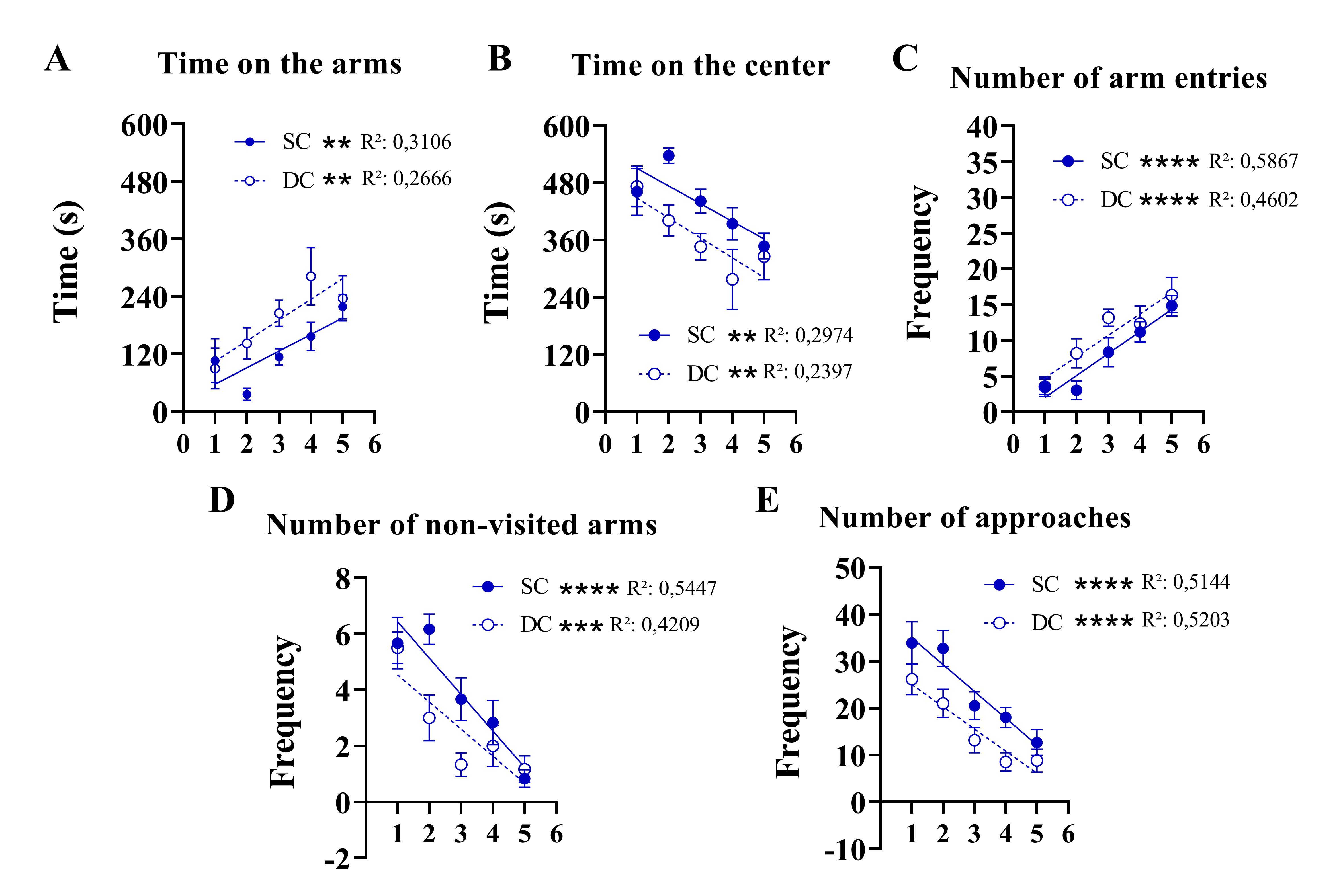

Supplement: Supplementary file 5 — Figure S5: Habituation in groups of different cages treated with ketamine: Starting on the 4th day of the assay, the 3DM test was performed once daily for 5 days. The test spent 10 min per session and was performed 20 min after the administration of ketamine (25 mg/kg). (A) habituation over time on the arms of KET‐C groups. (B) Habituation over time on the center of KET‐C groups. (C) Habituation over the number of arm entries on KET‐C groups. (D) Habituation over the number of non‐visited arms on KET‐C groups. (E) Habituation over the number of approaches on KET‐C groups. Same dyad along the test (SC), different dyad along the test (DC). For all groups, habituation in the KET‐C groups was analyzed using linear regression between the parameters and the sessions. All data were expressed as mean ± SEM **p < 0.01; ***p < 0.001; ****p < 0.0001. Next to the legends are the respective p and R 2 values. N = 6 for all groups. [file EJN-62-0-s006.tif]
