# Supplementary material for: Conspecific Presence Promotes Social Buffering, Restores Social Reward, and Enhances Spatial Navigation in a Ketamine‐Induced Model of Schizophrenia in Mice
Source: Eur J Neurosci. 2025 Dec 17;62(12):e70359. doi: 10.1111/ejn.70359 (PMC12710120; doi:10.1111/ejn.70359)
Supplement: Supplementary file 6 — Table S1: Correlation between 3DM parameters. [file EJN-62-0-s003.docx]

**Table S1. Correlation between 3DM parameters**

| **Pearson correlation** | Time on the arms | Time on the center | Number of arm entries | Number of non-visited arms | Number of approaches |
| --- | --- | --- | --- | --- | --- |
| Time on the arms | **1** |  |  |  |  |
| Time on the center | -0,98211 | **1** |  |  |  |
| Number of arm entries | 0,567495 | -0,6235 | **1** |  |  |
| Number of non-visited arms | -0,70488 | 0,75073 | -0,82115 | **1** |  |
| Number of approaches | -0,75238 | 0,756301 | -0,64686 | 0,7441 | **1** |

All correlations are significant (*p* < 0,05) and the values represent the R.
